# Anti-Inflammatory Effects and Mechanisms of Dandelion in RAW264.7 Macrophages and Zebrafish Larvae

**DOI:** 10.3389/fphar.2022.906927

**Published:** 2022-08-25

**Authors:** Wenju Li, Fulong Luo, Xiaohui Wu, Bei Fan, Mingran Yang, Wu Zhong, Dongyan Guan, Fengzhong Wang, Qiong Wang

**Affiliations:** ^1^ Institute of Food Science and Technology, Chinese Academy of Agricultural Sciences, Beijing, China; ^2^ Department of Emergency Medicine, The Affiliated Hospital of Southwest Medical University, Luzhou, China; ^3^ Department of Neurology, The First Affiliated Hospital, Chongqing Medical University, Chongqing, China; ^4^ Sichuan Provincial Rehabilitation Hospital, Chengdu, China; ^5^ Sino-Portugal TCM International Cooperation Center, The Affiliated Traditional Chinese Medicine Hospital of Southwest Medical University, Luzhou, China

**Keywords:** dandelion extract (DE), RAW264.7 cells, zebrafish larvae, M1/M2 subtype, inflammation, apoptosis

## Abstract

Dandelions (*Taraxacum* spp.) play an important role in the treatment of inflammatory diseases. In this study, we investigated the anti-inflammatory effects of Dandelion Extract (DE) in LPS-induced RAW264.7 macrophages and copper sulfate (CuSO_4_)-induced zebrafish larvae. DE was not toxic to RAW264.7 cells at 75 μg/ml as measured by cell viability, and DE inhibited LPS-induced cell morphological changes as measured by inverted microscopy. In survival experiments, DE at 25 μg/ml had no toxicity to zebrafish larvae. By using an enzymatic standard assay, DE reduced the production of nitric oxide (NO) in LPS-induced RAW264.7 cells. Fluorescence microscopy results show that DE reduced LPS-induced ROS production and apoptosis in RAW264.7 cells. DE also inhibited CuSO4-induced ROS production and neutrophil aggregation in zebrafish larvae. The results of flow cytometry show that DE alleviated the LPS-induced cell cycle arrest. In LPS-induced RAW264.7 cells, RT-PCR revealed that DE decreased the expression of M1 phenotypic genes iNOS, IL-6, and IL-1β while increasing the expression of M2 phenotypic genes IL-10 and CD206. Furthermore, in CuSO4-induced zebrafish larvae, DE reduced the expression of iNOS, TNF-α, IL-6, and IL-10. The findings suggest that DE reduces the LPS-induced inflammatory response in RAW264.7 cells by regulating polarization and apoptosis. DE also reduces the CuSO4-induced inflammatory response in zebrafish larvae.

## Introduction

Dandelions (*Taraxacum* spp.) are widely used in China, the Middle East, Western Europe, and America. There are 3,529 species of the genus dandelion, of which 2,336 species are named (Martinez et al., 2015; Sharifi-Rad et al., 2018). The most important species are *T. officinale* and *T. mongolicum*, which have very similar phytochemical profiles (Table 1), and both are used for similar indications, mainly liver and digestive disorders, and anti-inflammatory conditions including gout (Martinez et al., 2015; Sharifi-Rad et al., 2018). *T. officinale* is the main species used in traditional Chinese medicine in China, and was included in the first Chinese Pharmacopoeia in 1963. Dandelions extracts elicit their effects *via* various mechanisms, due to the presence of different classes of constituents with known anti-inflammatory effects.

**TABLE 1:** Important constituents reported in Dandelion extracts.

Constituent	*Taraxacum officinale*	*Taraxacum mongolicum*
Flavonoids		
Apigenin	+	+
Hesperidin	+	+
Isoetin	+	+
Luteolin	+	+
Quercetin	+	+
Phenolic acid derivatives		
Caffeic	+	+
Caffeoylquininic	+	+
Chlorogenic	+	+
Ferulic	+	+
Gallic	+	+
Coumarins		
Coumestrol		
Scopoletin	+	−
Heptaerythrolactone	+	+
Umbelliferolactone	+	−
Sesquiterpene lactones	+	+
Taraxacin		+
Sterols		
Lupeol	+	−
Sitosterol	+	−
Taraxasterol	+	−

Note: (+) detected, (-) not detected.

Previous anti-inflammatory studies on dandelions extracts have shown *T. mongolicum* extract inhibited nitric oxide production in activated RAW264.7 cells (Kim et al., 2011), and *T. officinale* extracts have significant anti-inflammatory and antioxidant effects in mouse inflammation models, inhibiting the production of cytokines (NO, PGE2, IL-1β, IL-6, and TNF-α, etc.) associated with inflammatory responses through the NF-κB pathway and MAPK pathway (Park et al., 2011; Kikuchi et al., 2016). Immunomodulation plays an important role in the inflammatory process and its intrinsic mechanism may be related to the balance of M1 and M2 phenotypes of macrophages. However, the effects on macrophage apoptosis and polarization by *T. officinale* have not previously been investigated.

Inflammation is a defensive immune response triggered by stimuli such as bacterial or viral infection, noxious stimuli, or cellular damage (Turner et al., 2014). Macrophages play an immune defense function in the inflammatory response *via* plasticity and functional polarization (Davies et al., 2013), and macrophages can undergo transformation of M1 and M2 phenotypes in specific microenvironments, and lipopolysaccharide (LPS) is a major component of the cell wall of Gram-negative bacteria that can mimic the early stages of the inflammatory response. LPS has the ability to polarize macrophages to the M1 phenotype, also known as classically active macrophages. M1 macrophages are capable of pro-inflammatory responses and release nuclear factor-κB (NF-κB) signaling pathway through activation of toll-like receptor (TLR) 4 mediated NF-κB signaling pathway to release pro-inflammatory mediators and cytokines such as inducible nitric oxide synthase (iNOS), tumor necrosis factor-α (TNF-α), interleukin-12 (IL-12), IL-6, IL-1β, etc. (Yi, 2016; Hernandez et al., 2019), which trigger type I immune response by boosting their own antigen-presenting capabilities, causing tissue cell injury to end the inflammatory response process. In contrast, interleukin 4 (IL-4) polarize macrophages to the M2 phenotype, also known as alternatively activated macrophage. and M2 macrophages are capable of pro-inflammatory responses and release such as IL-10, IL-4, arginine kinase -1 (Arginase-1), CD206, etc., which promote the self-repair process of tissue damage by inhibiting the development of the inflammatory response (Sica and Mantovani, 2012; Daniel et al., 2018). Thus, in infected tissues, macrophages are first polarized into the M1 phenotype that promotes the inflammatory response to help the host fight the pathogen. Subsequently, macrophages are polarized into an M2 phenotype with anti-inflammatory effects to repair damaged tissues.

Furthermore, excessive generation of reactive oxygen species (ROS), a key signaling molecule for defensive responses, can result in inflammatory responses and disease development (Bjørn and Hasselbalch, 2015; Liu et al., 2018), and ROS can control the apoptotic process (Walls et al., 2011). Stimulation of macrophages with LPS or zebrafish larvae with CuSO4 increases ROS production, increasing inflammation, whereas overproduction of inflammatory factors may cause excessive ROS production (Bjørn and Hasselbalch, 2015; Lanzarin et al., 2021).

To investigate the intrinsic mechanism of the anti-inflammatory effect of Dandelion (*T. officinale*) extract (DE) in macrophages, we used an LPS-induced *in vitro* culture of RAW264.7 macrophages to investigate any potential inhibitory effect of DE on LPS-induced macrophage ROS and apoptosis, as well as M1/M2 phenotype-related marker genes. To corroborate this approach, we employed transgenic zebrafish larvae to investigate the effects of DE on CuSO4-induced inflammation, measuring neutrophil aggregation, ROS generation, and inflammatory gene expression.

## Materials and Methods

### Materials

Dulbecco’s modified Eagle’s medium (DMEM) was purchased from Thermo Scientific; fetal bovine serum (FBS) from Gibco; Lipopolysaccharides (LPS) from Sigma; Nitric Oxide Assay Kit, Hoechst33342 from Beyotime; DCFH-DA Reactive Oxygen ROS Fluorescent Probe, MTT Kit from Solarbio; Cell Cycle Analysis Kit (with RNase) from 4A Biotech Co., Ltd.; EASYspinPlus rapid tissue/cellular RNA extraction kit from Aidlab; GoScript Reverse Transcription System from Promega; NovoStart SYBR qPCR SuperMix Plus from Novoprotein; and RT-PCR primers from Beijing Jingzhe Yongxing Biotechnology Co., (all Beijing, China).

### Plant Material and Preparation of the Extract

*T. officinale* of Chinese Pharmacopoeial quality (2020) was purchased from Beijing Taiyang Shukang Pharmaceutical Co. (Beijing, China). 0.5 Kg of herb was exhaustively extracted with boiling water and the decoction from two successive extractions collected. The filtrate was collected, spray dried into powder and stored at −20°C. The yield of extract was 18.8% (w/w).

### Phytochemical Analysis of Dandelion Extract

Ultra-performance liquid chromatography (UPLC-QE-Orbitrap-MS) was used to analyse the major constituents. Samples of DE were dissolved in water, vortexed for 30 s, sonicated for 30 min, centrifuged at 12,000 rpm at 4°C for 15 min and the supernatant filtered prior to the UPLC-QE-Orbitrap-MS analysis. Conditions were as follows: column, Waters’ BEH C18 column (1.7 m × 2.1 × 100 mm). Mobile phase A (aqueous formic acid solution 0.1%) and B (acetonitrile solution of formic acid: 0.1%), in a multi-step linear elution gradient program. A Q Exactive Focus mass spectrometer coupled with the Xcalibur software was employed to obtain the MS and MS/MS data based on the IDA acquisition mode.

### Cell Culture and Sample Treatment

The RAW264.7 murine macrophage cell line was obtained from Peking University School of Medicine (Beijing, China). Cells were grown in DMEM medium containing 10% fetal bovine serum, 5% CO2, at 37°C.cells were passaged every 24 h. Logarithmic growth phase cells were taken for experiments and inoculated at a density of 1×10^4^ cells/ml in 96-well plates at 100 μl per well, or at a density of 1×10^6^ cells/ml in 6-well plates at 2 ml per well. After 24 h, the experiment was performed with a control group, an LPS group (500 ng/ml), and a DE group (3.13, 6.25, 12.5, 25, and 50 μg/ml). DE was added to pre-activate for 2 h, and then 500 ng/ml of LPS solution was added to each well to co-stimulate for 36 h.

### Cell Viability Assay

RAW264.7 cells were cultured in the 96-well plates at a density of 1 × 10^4^ cells/well overnight and then exposed to various concentrations of DE and LPS (500 ng/ml) alone or in combination for 36 h. An MTT solution (0.5 mg/ml) were added to each well for another 4 h. The culture medium was removed and replaced by 100 μl dimethyl sulfoxide, and the absorbance was read at 490 nm with an enzyme marker.

### Cell Morphology

RAW264.7 cells were cultured overnight at a density of 1×10^4^ cells/well in 96-well plates and then exposed to different concentrations of DE and LPS (500 ng/ml) individually or in combination for 24 h. The morphological changes of each group were observed by inverted microscopy.

### Generation of Nitric Oxide

RAW264.7 cells were cultured overnight in 96-well plates at a density of 1×10^4^ cells/well, and then exposed to different concentrations of DE and LPS (500 ng/ml) individually or in combination for 24 h. 50 μl of the supernatant of each group of cells was added to 50 μl each of Griess Reagent I and Griess Reagent II solutions, respectively, and the absorbance of each group was measured using an enzyme marker. The absorbance of each group was measured at 540 nm.

### Reactive Oxygen Species and Apoptosis

RAW264.7 cells were cultured overnight at a density of 1×10^6^ cells/well in 6-well plates, then exposed to different concentrations of DE and LPS (500 ng/ml) alone or in combination, then exposed to different concentrations of DE and LPS (500 ng/ml) alone or in combination, ROS production was measured with the DCFH-DA Reactive Oxygen ROS Fluorescent Probe according to the manufacturer’s instructions, 1 ml of diluted DCFH-DA or Hoechst33342 solution was added at a dilution ratio of 1:1,000, and the final concentration was 10 μmol/L. The cells were incubated at 37°C for 30 min and washed. Cell morphology was observed by fluorescence microscopy and analyzed by Image-Pro Plus software (Wayne Rasband, National Institute of Health, Bethesda, MD, United States) and fluorescence intensity by flow cytometry after dilution with Hoechst33342.

### Cell Cycle Arrest

RAW264.7 cells were cultured overnight, then exposed to different concentrations of DE and LPS (500 ng/ml) alone or in combination. The cell cycle is measured by the Cell Cycle Analysis Kit according to the manufacturer’s instructions, cell cycle was detected by flow cytometry and analyzed by FlowJo software.

### Zebrafish Maintenance and CuSO_4_ Culture

Transgenic strains of zebrafish were obtained from the Zebrafish Resource Center in Wuhan, China. According to the Guidelines for the Testing of Chemicals (Effects on Biotic Systems, China), the zebrafish were reared under a 14 h light/10 h dark cycle at 28.5 ± 1.0°C. Zebrafish embryos were collected by natural spawning using Holfreter water (0.05 g/L K/Cl, 0.1 g/L CaCl_2_, 0.2 g/L NaHCO_3_, 3.5 g/L NaCl, pH 7.0) for storage. The 3 days post-fertilized (dpf) zebrafish embryos were randomly transferred to 6-well plates (30 tails per well) and divided into control, CuSO_4_ (10 µM) and DE (3.13, 6.25, 12.5, and 25 µg/ml). Zebrafish embryos were pretreated with different concentrations of DE for 1 h and then incubated in a mixture of DE and CuSO_4_ (10 µM) for 40 min.

### Neutrophils and Reactive Oxygen Species in Zebrafish Larvae

Zebrafish larvae (3 dpf) were randomly transferred into 6-well plates (20 strips per well) for neutrophil recruitment and ROS production studies. According to the Guidelines for the Testing of Chemicals (Effects on Biotic Systems, China), Embryos were pretreated with embryo medium dissolved in DE (3.13, 6.25, 12.5, and 25 μg/ml) for 1 h, incubated in a mixture of DE and CuSO_4_ (10 μM) for 40 min, rinsed and anesthetized with MS222. Larvae were photographed under a body vision fluorescence microscope to observe neutrophil recruitment, ROS production was measured with the DCFH-DA Reactive Oxygen ROS Fluorescent Probe according to the manufacturer’s instructions, intensity was analyzed by Image-Pro Plus software.

### RNA Isolation and Quantitative Real-Time Reverse Transcriptase Polymerase Chain Reaction

Cellular and zebrafish larval RNA were obtained according to the EASYspinPlus rapid tissue/cellular RNA extraction kit. The cDNA was synthesized by reverse transcription of the first strand according to the GoScript Reverse Transcription System. The oligonucleotide primers for iNOS designed from mouse were GAG​GCC​CAG​GAG​GAG​AGA​GAT​CCG (forward) and TCC​ATG​CAG​ACA​ACC​TTG​GTG​TTG (reverse), for IL-1β designed from mouse were AAA​TAC​CTG​TGG​CCT​TGG​GC (forward) and CTT​GGG​ATC​CAC​ACT​CTC​CAG (reverse), for IL-6 designed from mouse were CCA​GAG​ATA​CAA​AGA​AAT​GAT​GG (forward) and ACT​CCA​GAA​GAC​CAG​AGG​AAA​T (reverse), for IL-10 designed from mouse were GTG​GAG​CAG​GTG​AAG​AGT​GA (forward) and TCG​GAG​AGA​GGT​ACA​AAC​GAG (reverse), for CD206 designed from mouse were CTT​CGG​GCC​TTT​GGA​ATA​AT (forward) and TAG​AAG​AGC​CCT​TGG​GTT​GA (reverse), for Arginase-1 designed from mouse were GTG​AAG​AAC​CCA​CGG​TCT​GT (forward) and GTG​AAG​AAC​CCA​CGG​TCT​GT (reverse), for β-actin designed from mouse were GGC​TGT​ATT​CCC​CTC​CAT​CG (forward) and CCA​GTT​GGT​AAC​AAT​GCC​ATG​T (reverse), for iNOS designed from zebrafish CCC​GTG​TTC​CAC​CAG​GAG​AT (forward) and CCC​GTG​TTC​CAC​CAG​GAG​AT (reverse), for TNF-α designed from zebrafish TAT​CAG​ACA​ACC​GTG​GCA​CC (forward) and GCT​TCA​GCA​CTT​TTC​CGT​GG (reverse), for IL-6 designed from zebrafish GCA​GTA​TGG​GGG​AAC​TAT​CCG (forward) and GCA​GTA​TGG​GGG​AAC​TAT​CCG (reverse), for IL-10 designed from zebrafish TCC​ACA​ACC​CCA​ATC​GAC​TC (forward) and AAG​AGC​AAA​TCA​AGC​TCC​CCC (reverse), for GAPDH designed from zebrafish TGA​AAT​TGC​CGC​ACT​GGT​TG (forward) and AGC​CTC​ATC​ACC​AAC​GTA​GC (reverse). Steadystate mRNA levels of iNOS, IL-1β, IL-6, IL-10, CD206, Arginase-1, TNF-α, GAPDH and β-actin were determined by quantitative PCR using RT-PCR amplifiers (ABI,United States). A dissociation curve analysis of target mRNAs showed a single peak for each. The mean Ct of the gene of interest was calculated from triplicate measurements and normalized with the mean Ct of a control gene GAPDH or β-actin.

### Statistical Analysis

GraphPad Prism8.0 software (GraphPad Software, Inc., La Jolla, CA, United States) was used for statistical analyses. Results were expressed as mean ± standard deviation (x̅ ± s), *t*-test was used to compare the differences between two groups, and a one-way analysis of variance (ANOVA) was used to compare the differences between multiple groups, with *p* < 0.05 being a significant difference and *p* < 0.01 being a highly significant difference.

## Results

### Phytochemical Identification of EPS by UHPLC-QE-MS

In positive and negative flow modes, 406 potential DE components were discovered, with the most important presented in Table 2.

**TABLE 2:** Characterization of chemical constituents in DE by UHPLC-QE-MS analysis.

NO.	Name	RT (min)	InChIKey	Formula	molecular mass	Class
1	Gallic acid	1.07	LNTHITQWFMADLM-UHFFFAOYSA-N	C7H6O5	170.12	Phenols
2	Protocatechualdehyde	2.66	IBGBGRVKPALMCQ-UHFFFAOYSA-N	C7H6O3	138.12	Phenols
3	Phenylacetic acid	2.86	WLJVXDMOQOGPHL-UHFFFAOYSA-N	C8H8O2	136.15	Aromaticity
4	Caftaric acid	2.87	SWGKAHCIOQPKFW-JTNORFRNSA-N	C13H12O9	312.23	Phenylpropanoids
5	Chlorogenic acid	2.94	CWVRJTMFETXNAD-JUHZACGLSA-N	C16H18O9	354.31	Phenylpropanoids
6	Ferulic acid	3.36	KSEBMYQBYZTDHS-HWKANZROSA-N	C10H10O4	194.18	Phenylpropanoids
7	Apigenin-8-C-glucoside	5.99	SGEWCQFRYRRZDC-VPRICQMDSA-N	C21H20O10	432.38	Flavonoids
8	3,5-Dicaffeoylquinic acid	6.09	KRZBCHWVBQOTNZ-RDJMKVHDSA-N	C25H24O12	516.45	Phenylpropanoids
9	Liquiritigenin	6.90	FURUXTVZLHCCNA-AWEZNQCLSA-N	C15H12O4	256.25	Flavonoids
10	Luteolin	7.08	IQPNAANSBPBGFQ-UHFFFAOYSA-N	C15H10O6	286.24	Flavonoids
11	Quercetin	7.10	REFJWTPEDVJJIY-UHFFFAOYSA-N	C15H10O7	302.24	Flavonoids
12	Hesperetin	7.22	AIONOLUJZLIMTK-AWEZNQCLSA-N	C16H14O6	302.28	Flavonoids
13	Kaempferide	8.16	SQFSKOYWJBQGKQ-UHFFFAOYSA-N	C16H12O6	300.26	Flavonoids
14	Diosmetin	8.17	MBNGWHIJMBWFHU-UHFFFAOYSA-N	C16H12O6	300.26	Flavonoids

### Effect of Dandelion Extract on the Activity of RAW264.7 Cells

**FIGURE 1:**
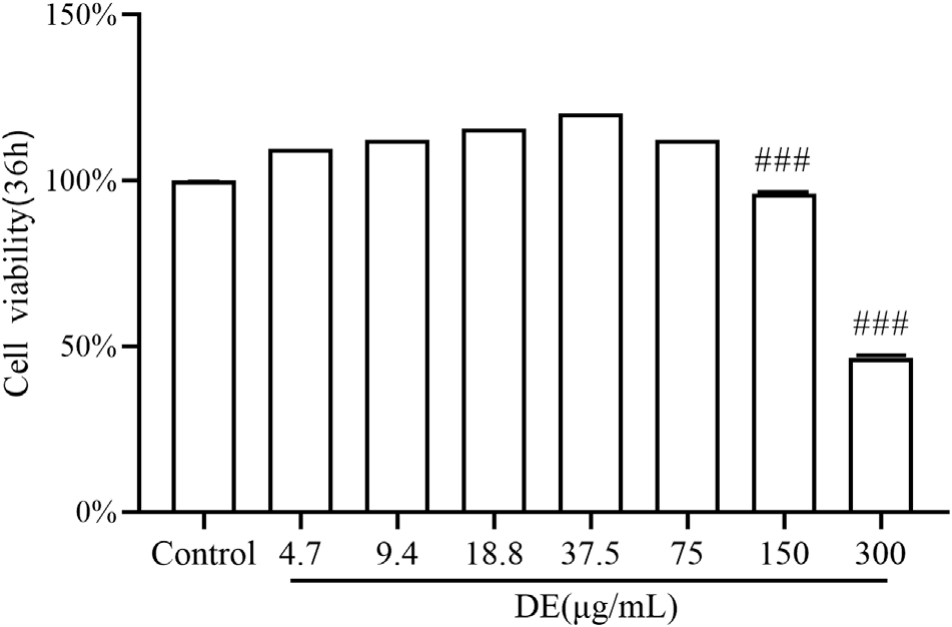
Effect of DE on the activity of RAW264.7 cells. Cells were treated with DE (0–300 μg/ml) for 36 h, and cell viability was measured by the cell viability assay. Compared with the control group,^###^ *p* < 0.001.

The cell viability assay findings (Figure 1) revealed that, compared to the control, the cell viability of DE at concentrations of 150 and 300 μg/ml was (96.23 ± 0.14)% and (46.46 ± 0.11)%, with concentration-dependent ;inhibition of cell proliferation. DE concentrations less than 75 μg/ml had no influence on cell multiplication or survival rate, hence a concentration range less than 75 μg/ml was employed in the following studies.

### Effect of Dandelion Extract on the LPS-Induced Morphology in RAW264.7 Cells

**FIGURE 2:**
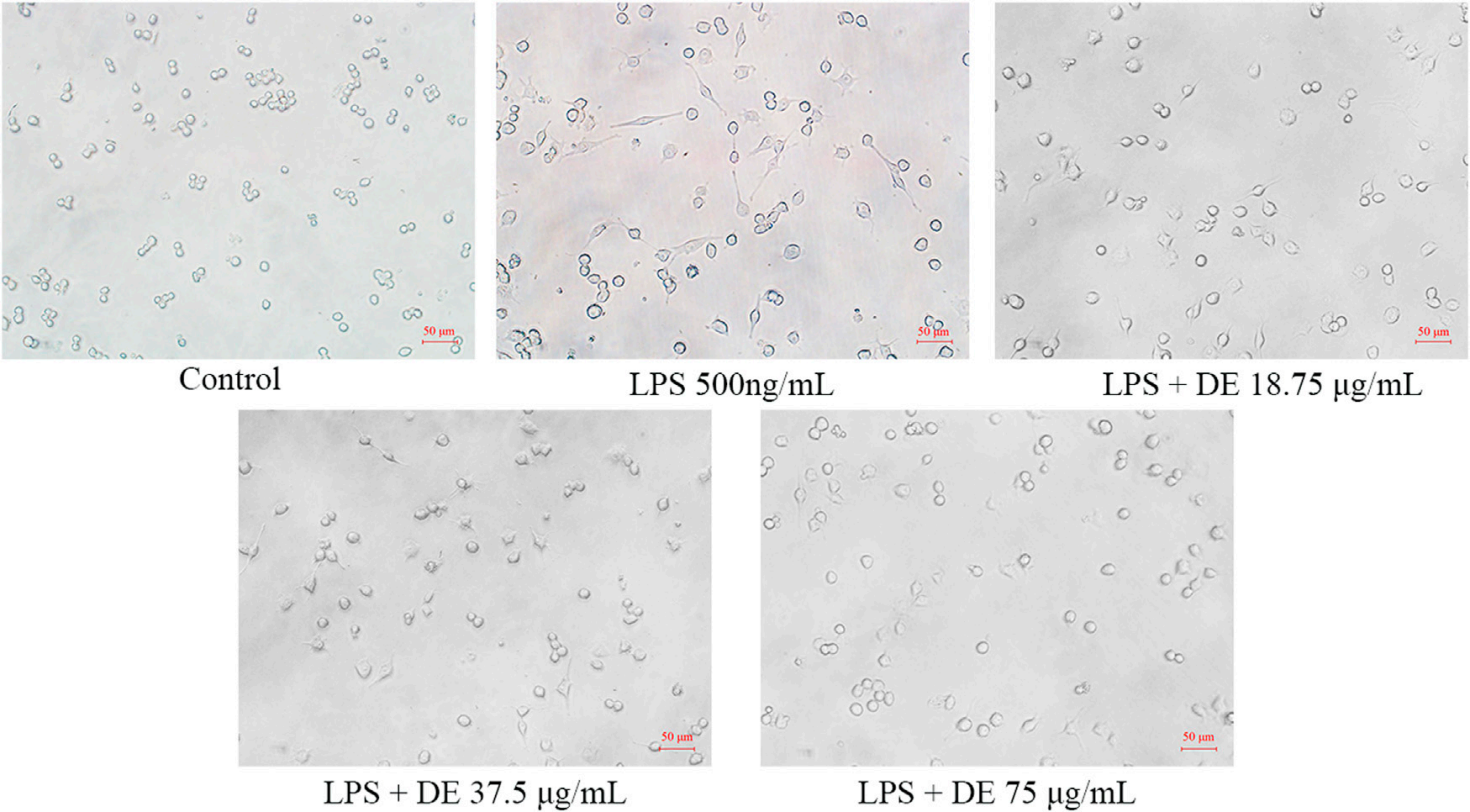
Effect of DE on the morphology of RAW264.7 cells after LPS induction (×200). Cells were pretreated with DE (18.75, 37.5, and 75 μg/ml) for 2 h and then stimulated with LPS (500 ng/ml) for another 24 h. Changes in cell morphology were observed with an inverted microscope.

The morphology of RAW264.7 cells changes with LPS stimulation (Tu et al., 2021). Figure 2 reveals that the control cells are irregularly circular, but the LPS cells are bigger, have irregular tentacles, and are shuttle-shaped. While LPS increased cell adhesion to the wall, the DE groups’ cell shape tended to be comparable to the control, with some cells returning to semi-adherent round.

### Dandelion Extract Reduced LPS-Induced Nitric Oxide Production in RAW264.7 Cells

**FIGURE 3:**
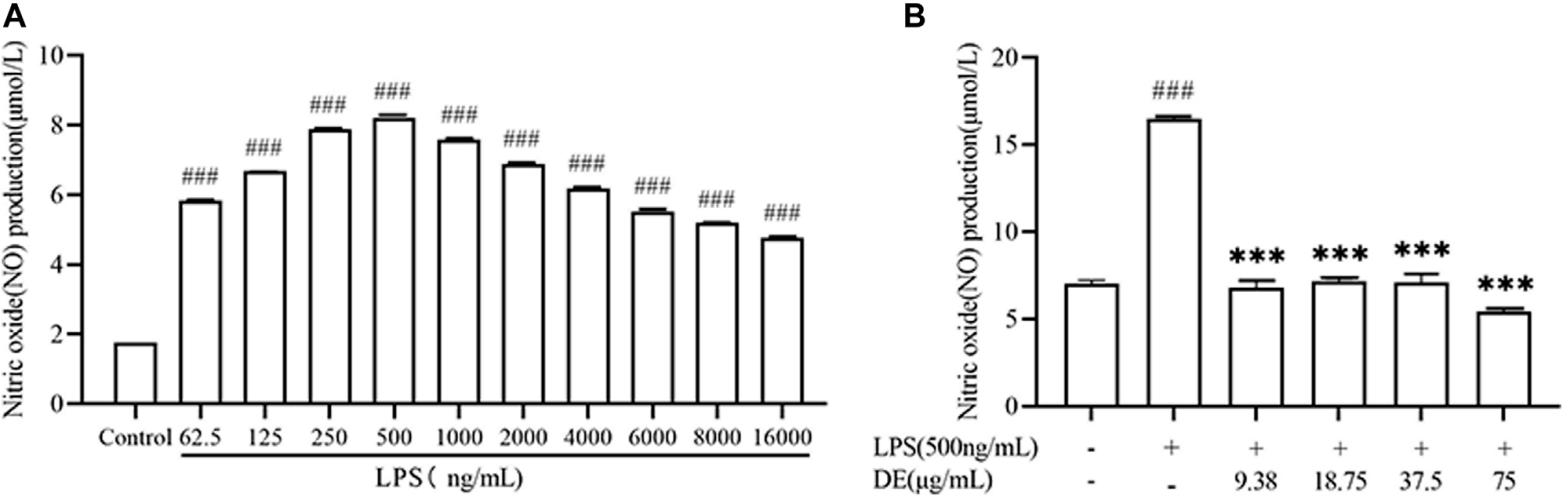
NO production after LPS stimulation and after treatment with DE. Cells were pre-stimulated with different concentrations of LPS for 24 h. Cell supernatant NO production was measured using an enzyme marker **(A)**. Cells were pretreated with DE for 2 h and then stimulated with LPS (500 ng/ml) for another 24 h. Cell supernatant NO production was measured using an enzyme marker **(B)**. Compared with the control group, ^###^ *p* < 0.001; compared with the LPS group,***p < 0.001.

The stimulation of RAW264.7 cells with LPS increased NO production. (Lin et al., 2021). Figure 3 A shows that, as compared to the control group, NO production was maximal at 500 ng/ml of LPS stimulated cells, and subsequently dropped as LPS concentration rose. The impact of DE on cellular NO generation was then studied in the presence of 500 ng/ml LPS. DE (9.38, 18.75, 37.5, and 75 μg/ml) inhibited the release of LPS-stimulated NO generation in Figure 3B.

### Effect of Dandelion Extract on LPS-Induced Intracellular Reactive Oxygen Species in RAW264.7 Cells

**FIGURE 4:**
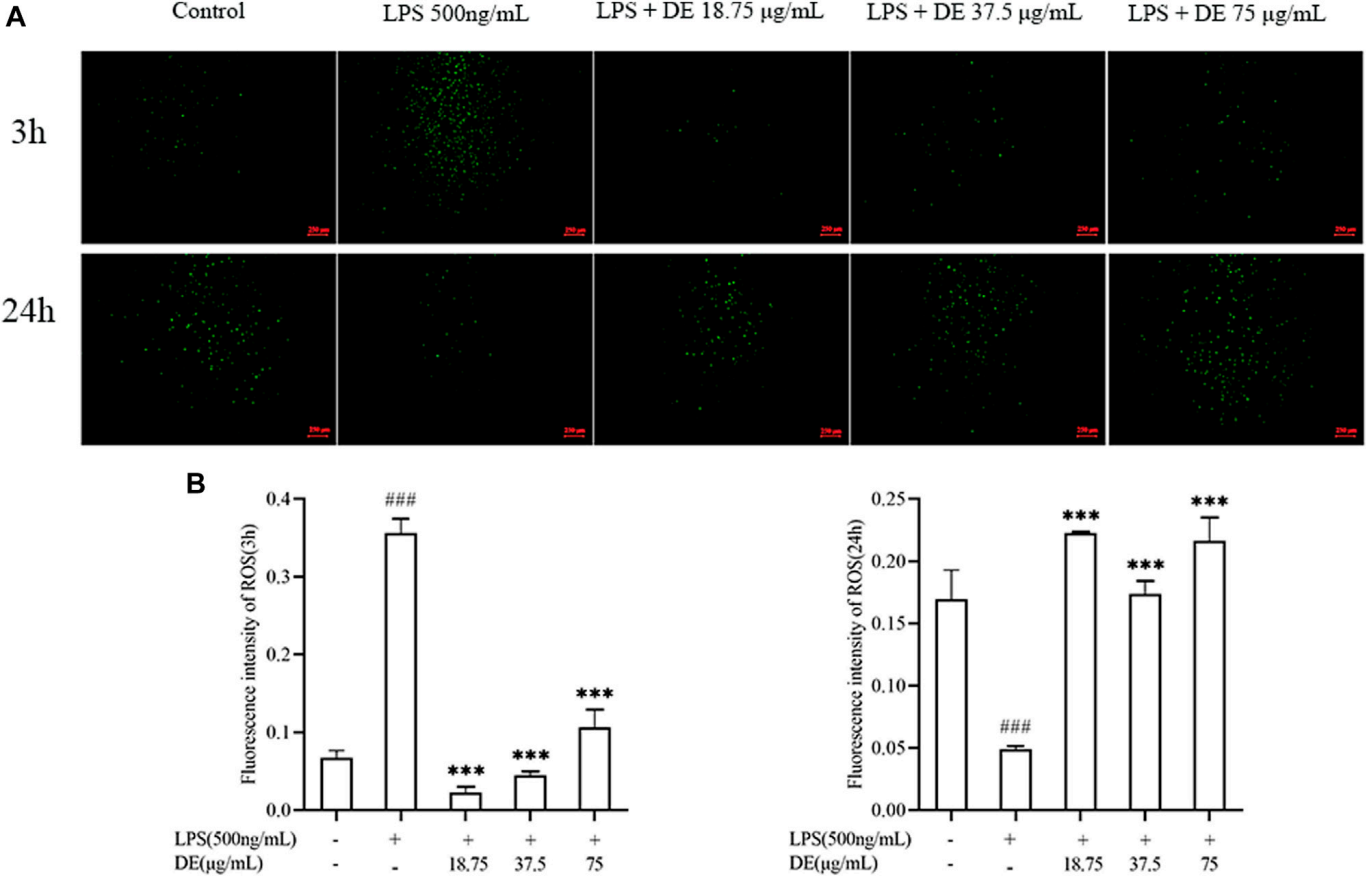
DE reduced ROS production after LPS stimulation (×40). Cells were pretreated with DE for 2 h and then stimulated with LPS (500 ng/ml) for another 3 h or 24 h. Fluorescence microscopy was used to take pictures **(A)** and the fluorescence intensity of ROS for 3 h **(B)** or 24 h **(C)** was obtained by analysis with Image J software. Compared with the control group, ^###^ *p* < 0.001; compared with the LPS group, ***p < 0.001.

As previously demonstrated, LPS stimulation increased ROS production in RAW264.7 cells (Liang et al., 2021). Figures 4A,B show the effect of 500 ng/ml LPS stimulation on RAW264.7 cells for 3 h. The LPS group’s ROS fluorescence intensity was substantially higher than the control, but DE (18.75, 37.5, and 75 μg/ml) reduced the LPS-induced intensity. Figures 4A,C illustrate the opposite result after 24 h of LPS stimulation. DE enhanced the intensity of LPS-induced fluorescence.

### Effect of Dandelion Extract on LPS-Induced Cell Cycle

**FIGURE 5:**
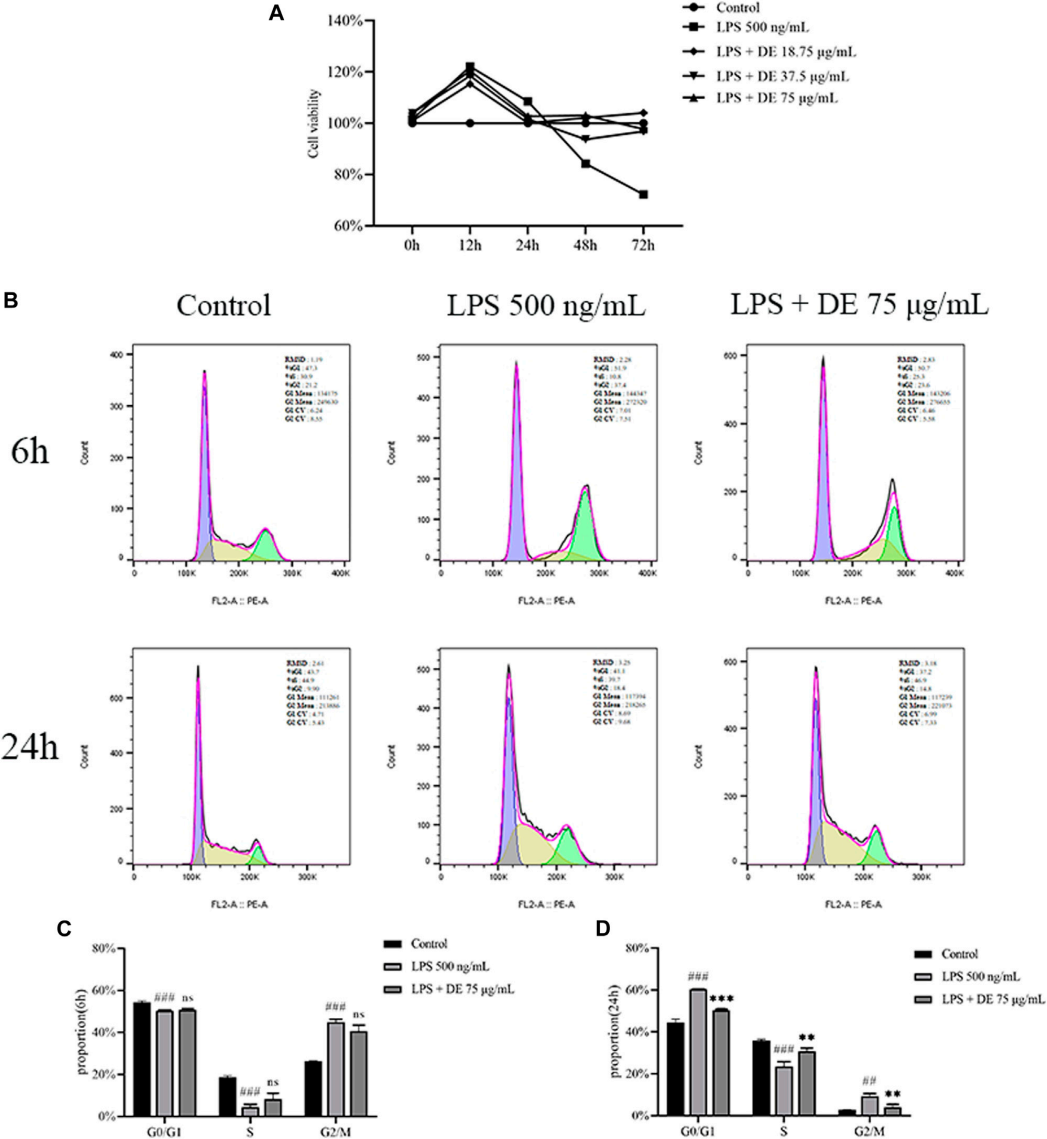
Effect of DE on the LPS-induced RAW264.7 cell cycle. Cells were treated with DE with or without LPS (500 ng/ml) co-treatment for 6 h, 12 h, 24 h, 48 h, 72 h, and the cell viability was measured by the cell viability assay **(A)**. The cell cycle was detected by flow cytometry at 6 h and 24 h **(B)**, and the cycle changes were analyzed at 6 h **(C)** and 24 h **(D)**. Compared with the control group, ^###^ *p* < 0.001; compared with the LPS group,***p < 0.001.

Figure 5A shows that DE decreased LPS-induced apoptosis in RAW264.7 cells. LPS and DE (18.75, 37.5, and 75 g/ml) co-stimulated RAW264.7 cells during 0–12 h, Cell viability increased significantly in the LPS group. The cell viability of the LPS group gradually reduced compared to the control at 12–72 h, however DE significantly increased the cell vitality of the LPS group after 24 h.

The results of flow cytometry (Figures 5B,C) show the effects of LPS, DE, and co-stimulated RAW264.7 cells for 6 h. The proportion of G0/G1 phase and S phase RAW264.7 cells were significantly reduced in the LPS group compared with the control, and the proportion of G2/M phase RAW264.7 cells was significantly increased, but there was no difference in the DE group compared with the LPS group. After co-stimulation for 24 h (Figures 5B,D), the proportion of G0/G1 and S phase cells in the LPS group was significantly decreased compared with the control. DE attenuated this increase. The proportion of G2/M phase cells in the LPS group was significantly increased, and again DE decreased this percentage.

### Effect of Dandelion Extract on LPS-Induced Apoptosis in RAW264.7 Cells

**FIGURE 6:**
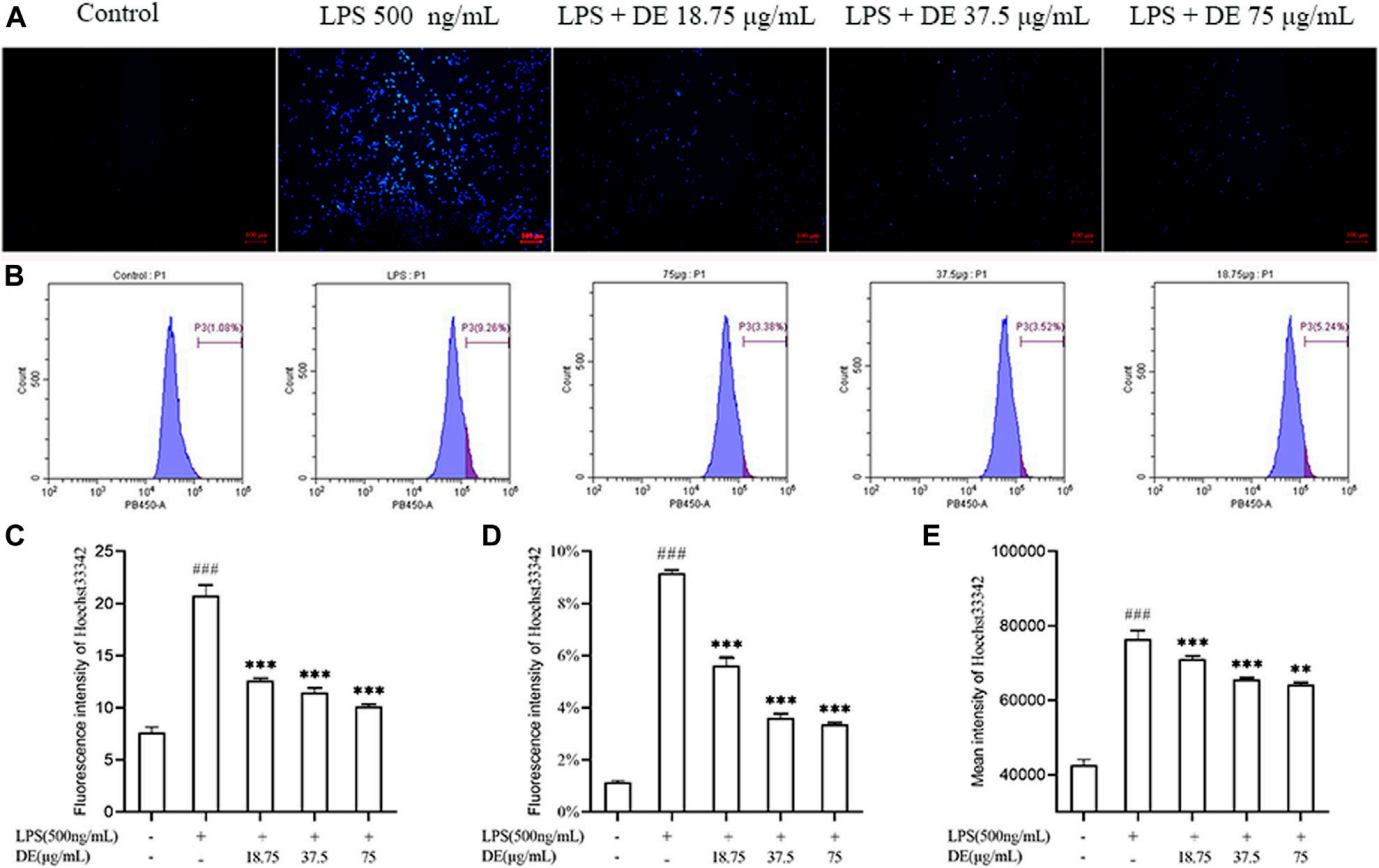
DE reduction of apoptosis in RAW264.7 cells after LPS stimulation (×100). Cells were pretreated with DE (18.75, 37.5, and 75 μg/ml) for 2 h and then stimulated with LPS (500 ng/ml) for another 3 h or 24 h. Cells were observed using a fluorescent microscope **(A)**; fluorescence intensity recorded by flow cytometry **(B)**; analysis of fluorescence intensity using Image J software**(C)**. Analysis of the proportion of fluorescence intensity **(D)** and the average fluorescence intensity **(E)**. Compared with the control group, ^###^ *p* < 0.001; compared with the LPS group, ***p < 0.001, **p < 0.01.

DE reduced LPS-induced apoptosis of RAW264.7 cells as shown in Figures 6A,C. Fluorescence intensity was increased in the LPS group, and this was reduced by DE. Flow cytometry (Figure 6B,D,E) showed that LPS stimulation increased the percentage of high fluorescence intensity cells and the average fluorescence intensity of cells, whereas DE decreased this compared to the LPS group.

### Effect of Dandelion Extract on LPS-Induced Polarisation of RAW264.7 Cells

**FIGURE 7:**
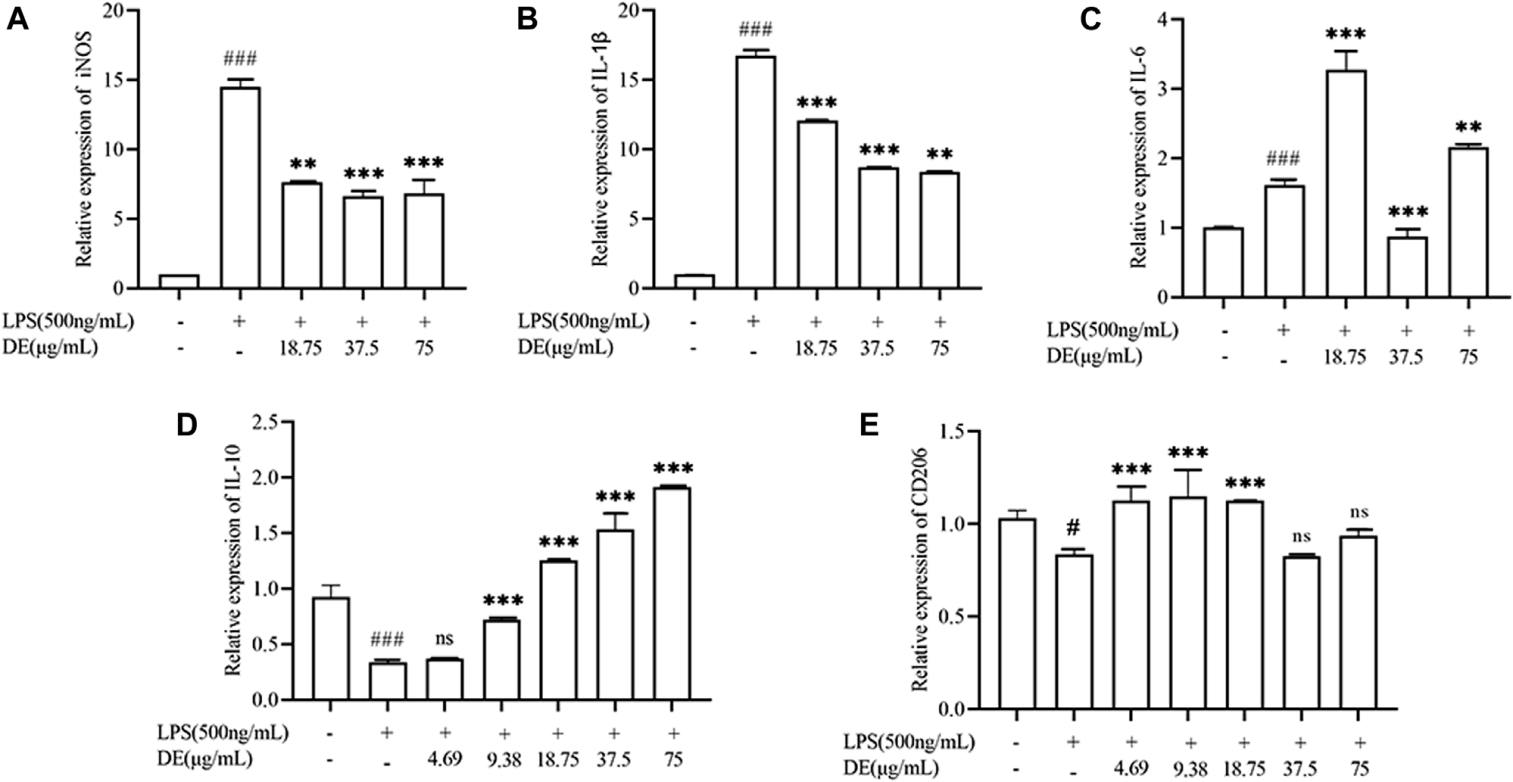
Modulation of LPS-stimulated polarization of RAW264.7 cells by DE. Cells were pretreated with DE (4.69, 9.38, 18.75, 37.5, and 75 μg/ml) for 2 h and then stimulated with LPS (500 ng/ml) for another 36 h. The expression levels of M1 phenotype genes (IL-1β, IL-6, and iNOS) **(A–C)**, and M2 phenotype genes (CD206, IL-10) **(D,E)** were obtained by RT-PCR. Compared with the control group, ^###^ *p* < 0.001, ^#^ *p* < 0.05; compared with the LPS group, ***p < 0.001, **p < 0.01.

We further investigated the regulatory effect of DE on cell polarization. The results in Figure 7 show that DE decreased LPS-induced up-regulation of M1 phenotype genes (IL-1β, IL-6, iNOS) mRNA expression, while increasing LPS-induced down-regulation of M2 phenotype genes (IL-10, CD206) mRNA expression.

### Dandelion Extract Reduces Neutrophil Aggregation and Reactive Oxygen Species Production in Transgenic Zebrafish Larvae

**FIGURE 8:**
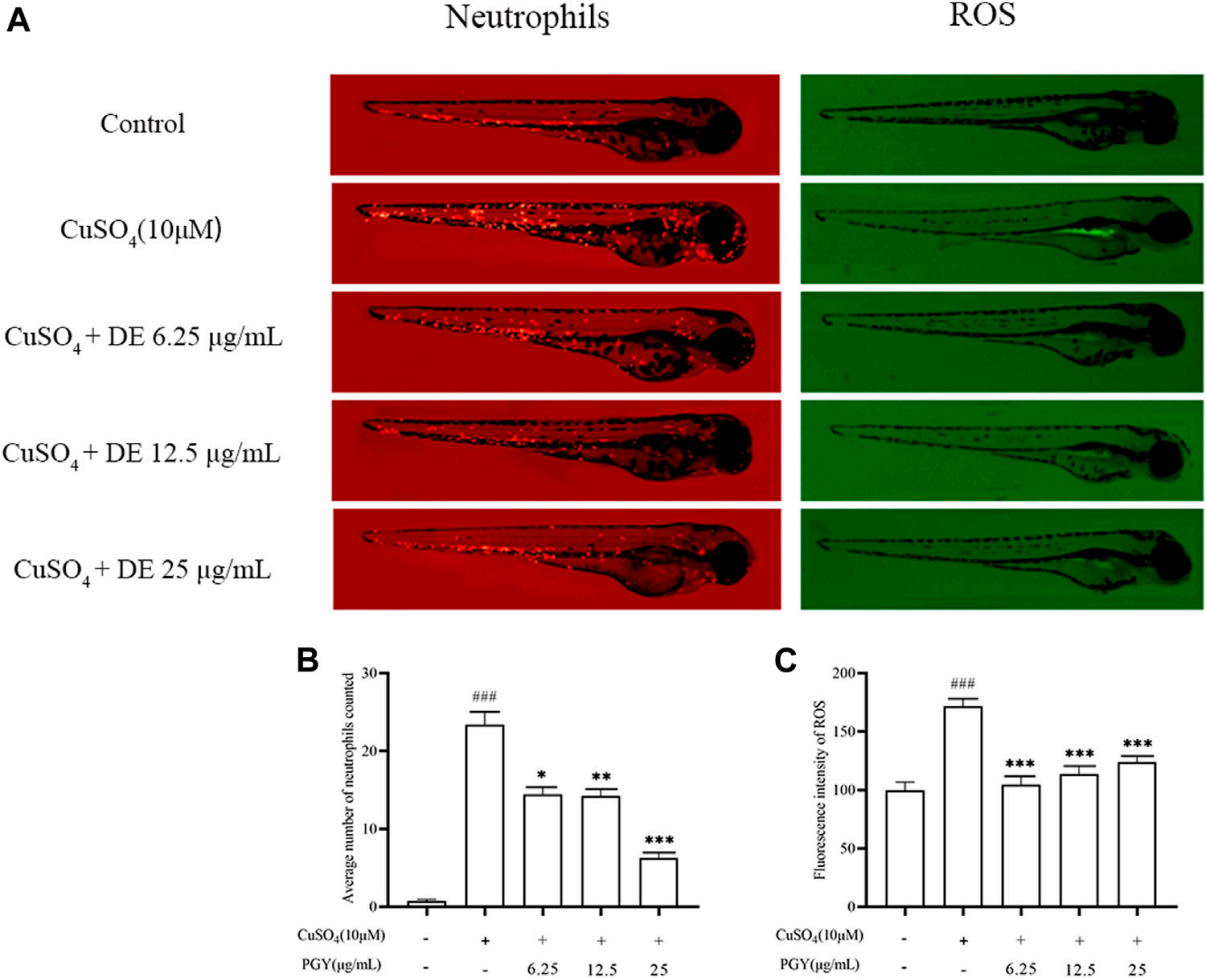
DE reduces neutrophil aggregation and ROS production in zebrafish larvae. Zebrafish larvae from 3 dpf were pretreated with DE for 1 h and then stimulated with CuSO_4_ (10 μM) for a further 40 min. Fluorescence intensity of neutrophils and ROS were recorded by fluorescence microscopy **(A)** and analyzed using Image J software for neutrophil aggregation **(B)** and ROS production **(C)**. Compared with the control group, ^###^ *p* < 0.001; compared with the CuSO_4_ group, ***p < 0.001, **p < 0.01.

In zebrafish larvae, CuSO_4_ has been shown to induce neutrophil recruitment and ROS production (Gong et al., 2020). Figures 8A,B show that DE (6.25, 12.5, and 25 µg/ml) reduced CuSO_4_ -induced neutrophil aggregation in zebrafish larvae. DE also reduced CuSO_4_ -induced ROS production (Figures 8A,C).

### Dandelion Extract Reduces the Expression of Inflammatory Genes in Zebrafish Larvae

**FIGURE 9:**
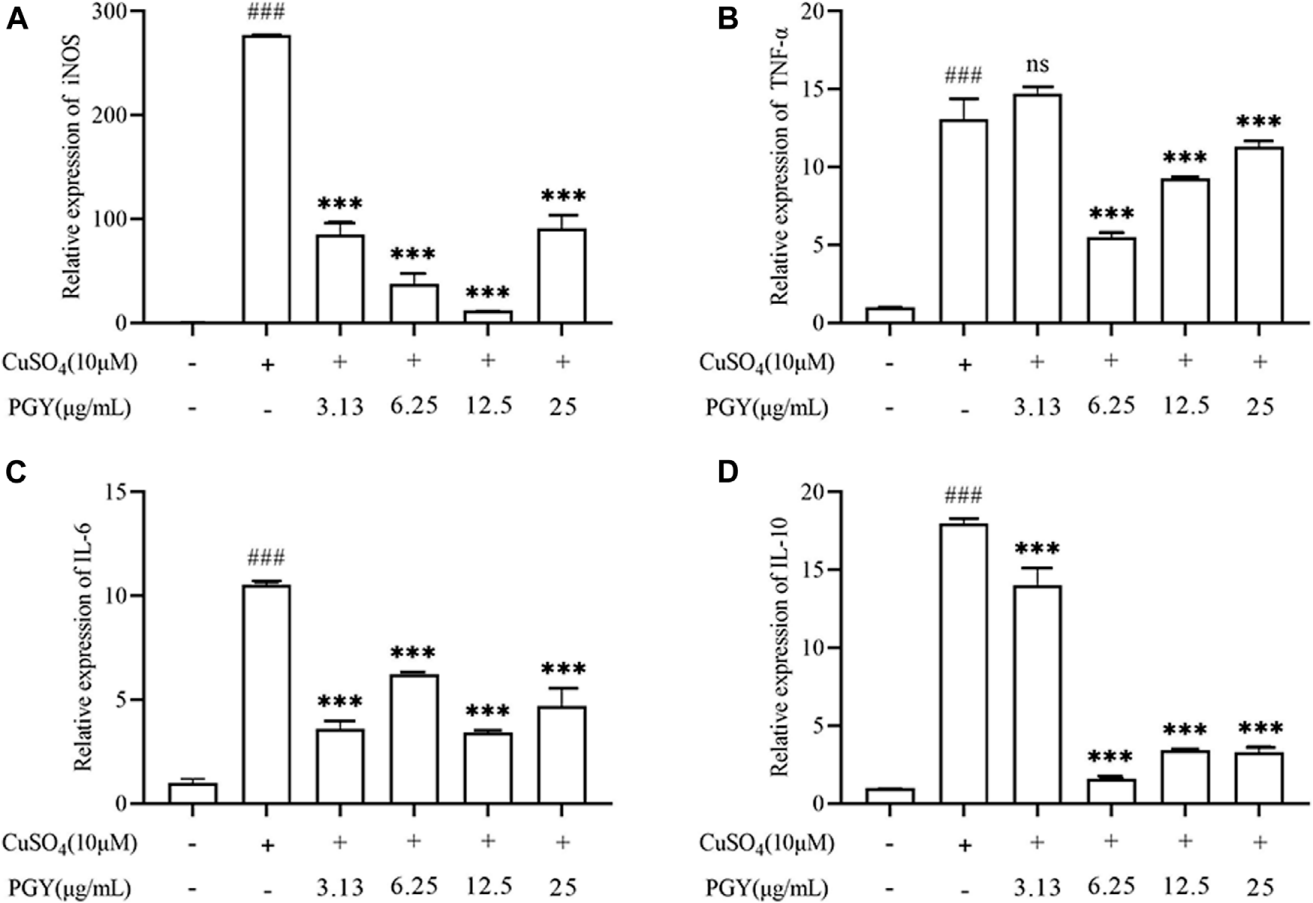
DE reduces the expression of inflammatory factors in zebrafish larvae. Zebrafish larvae from 3 dpf were pretreated with DE in culture for 1 h and then stimulated with CuSO_4_ (10 μM) for a further 40 min. The total mRNA was extracted and RT-PCR was performed to analyse iNOS, TNF-α, IL-6, and IL-10 gene expression. β-Actin was used as an internal control **(A–D)**. Compared with the control group, ^###^ *p* < 0.001; compared with the CuSO_4_ group, ***p < 0.001.

CuSO_4_ induced the expression of inflammatory mediators. increasing the expression of iNOS, IL-6, IL-10, TNF-α, while DE (3.13, 6.25, 12.5, and 25 µg/ml) inhibited the expression of these factors as shown in Figure 9.

## Discussion

Previous studies have shown that inflammatory responses are prevalent and involved in the development of diseases such as chronic obstructive pulmonary disease, inflammatory bowel disease, neurodegenerative diseases, cardiovascular diseases, digestive diseases, and diabetes (Liu et al., 2018; Aksentijevich et al., 2020). Inflammation not only reduces and eliminates damage caused by harmful factors, but also promotes late tissue repair. However, excessive or abnormal inflammation can lead to further damage to the organism. These inflammatory diseases impose a considerable economic burden on society and shorten the average life expectancy of patients. NSAIDs are commonly used as drugs to suppress inflammatory symptoms, and their long-term use can produce serious side effects, limiting their use (Utzeri and Usai, 2017; Ferrer et al., 2019). Therefore, there is an urgent need to study reliable and effective alternative drugs for the prevention and treatment of various diseases.

In the study, 406 compounds were identified with DE using UHPLC-QE-MS analysis. The main components reported on dandelion are triterpenoids, dandelionin, dandelion sterols, quercetin, lignan, chlorogenic acid, chicory acid, caffeotartaric acid, coumarin, etc. (Esatbeyoglu et al., 2017; Wang et al., 2020). *In vitro* experiments have shown that aqueous or alcoholic extracts of dandelion (lignan, chlorogenic acid, chicory acid, eucalyptolactones, etc.) can reduce the production of inflammatory factors (NO, PGE2, IL-1β, IL-6, and TNF-α, etc.) to suppress inflammation (Park et al., 2011; Kikuchi et al., 2016). Taraxerol or aqueous extracts (protocatechuic acid, caffeic acid, coumaric acid, ferulic acid, etc.) also improve the inflammatory symptoms of human umbilical vein endothelial cells by inhibiting the expression of NF-κB pathway and related inflammatory factors and adhesion factors, and it can be used to treat chronic diseases such as atherosclerosis and mastitis (Hu et al., 2017). *In vivo* tests, dandelion sterols were effective against alleviating inflammatory symptoms and reducing the production of pro-inflammatory factors in a mouse model of rheumatoid arthritis disease (Jiang et al., 2016; Chen et al., 2019), and these reports suggest that the inflammatory mediator inhibitory effects of DE may be at least partially due to the presence of these compounds. Further studies are needed to identify the active components in DE.

NO is known to be important in normal physiological conditions, and lipopolysaccharide (LPS) is a prominent component of Gram-negative bacteria’s cell wall that mimics the early phases of the inflammatory response. When activated by LPS, macrophages release pro-inflammatory mediators and cytokines *via* the toll-like receptor (TLR)4-mediated nuclear factor-B (NF-B) signaling pathway (Yi, 2016; Hernandez et al., 2019). However, increased iNOS expression catalyzes the generation of excess NO, and excess NO promotes inflammatory responses and tissue damage (Lee et al., 2017; Saini and Singh, 2019). As a result, LPS-induced NO production in macrophages may be used to evaluate the course of inflammation, and NO production inhibition may have an inhibitory effect on the inflammatory response. In the study, we discovered that DE reduced NO production as well as iNOS mRNA expression in cells following LPS stimulation, suggesting that DE may have a potential anti-inflammatory effect.

Reactive oxygen species (ROS), an important active component of the inflammatory response *in vivo*, is mainly produced by NADPH oxidase (Nox), and the cellular activity of ROS has a bidirectional effect and can amplify the secondary messengers of inflammation by activating downstream signaling cascade responses (Hong et al., 2008; Yang et al., 2013). In the study, LPS stimulated RAW264.7 cells to produce ROS at 3 h. DE reduced ROS production at 3 h of LPS stimulation. The nuclear transcription factor Nrf2 has been reported to play an important regulatory role in ROS production, and Nrf2 activity is inhibited at the very beginning of LPS stimulation of cells before it is activated (Liu et al., 2021). ROS production is likely to be regulated by Nrf2, with early LPS stimulation resulting in decreased Nrf2 production and increased ROS production, and as time passes, Nrf2 production rises and ROS production declines. In addition, ROS also activated the non-specific immune system to eliminate pathogens to suppress inflammation and repair damaged tissues (Blaser et al., 2016; Shen et al., 2020). Intracellular ROS production was reduced by LPS stimulation of cells for 24 h, whereas DE increased ROS production at 24 h of LPS stimulation. Furthermore, DE reduced copper sulfate-induced neutrophil aggregation in zebrafish larvae as well as ROS production in zebrafish larvae.

On the one hand, previous studies have found that ROS can regulate the apoptotic process (Nathan and Cunningham-Bussel, 2013). ROS exert a bidirectional regulatory effect on macrophages, with ROS both promoting and inhibiting macrophage apoptosis, which may be related to cAMP activation of cAMP-dependent protein kinases (Blaser et al., 2016). ROS production was significantly reduced at 24 h of LPS induction and apoptosis was significantly increased in RAW264.7 cells. DE increased ROS production at 24 h of LPS stimulation and reduced apoptosis at 24 h of LPS stimulation. DE exerts an anti-inflammatory effect probably by increasing ROS in macrophages and inhibiting apoptosis in their own cells, which in turn exerts an immunomodulatory function of macrophages.

It was found that LPS also mediates inflammatory responses through Toll-like receptor 4 (TLR4) (Huang et al., 2021). However, TLR4 activation effectively induces macrophage G0 phase arrest, and G0 phase arrest is accompanied by upregulation of p21 (Mlcochova, et al., 2020), an important transcriptional target of p53 that mediates DNA damage-induced macrophage cell cycle arrest (Besson et al., 2008), which regulates macrophage apoptosis. We found that LPS stimulation for 24 h induced macrophage G0/G1 phase block and DE relieved macrophage G0/G1 phase block, whereas LPS stimulation for 6 h failed to induce macrophage G0/G1 phase block, but promoted G2/M phase proliferation. The cell viability assay results demonstrated that LPS promoted cell proliferation within 24 h of LPS stimulation of RAW264.7 cells, and LPS promoted apoptosis after 24 h of LPS stimulation of RAW264.7 cells, and DE inhibited LPS-induced apoptosis of RAW264.7 cells, which may be a result of DE’s ability to deregulate the LPS-induced increase in P21 by downregulating macrophage G0/G1 phase block, thereby inhibiting apoptosis in macrophages, but further studies are needed to confirm this. In addition, the regulation of apoptosis by DE may be related to the deregulation of macrophage G0/G1 phase block by ROS through the upregulation of P21 expression by cAMP, but further studies are needed.

The polarization of macrophages is involved in the regulation of various disease processes, such as immune neuritis, inflammatory bowel disease (Harusato et al., 2013), diabetes and obesity (Feng et al., 2011), rheumatoid arthritis (Wang et al., 2017), etc. Macrophage polarization is induced by LPS to M1-type or by IL-4 to M2-type macrophages (Kimbrough et al., 2018). Our previous study established a model of LPS-induced polarization of RAW264.7 cells (results not shown), and DE significantly decreased the level of LPS-induced M1 phenotype IL-1β, iNOS, and IL-6 mRNA expression in RAW264.7 cells, while DE significantly increased the level of M2 phenotype CD206 and IL-10 mRNA expression to exert anti-inflammatory effects. However, the link between M1/M2 phenotype transformation and apoptosis in RAW264.7 cells needs to be further investigated.

CuSO_4_-stimulated zebrafish model acts as an *in vivo* model of inflammation exhibiting inflammatory response similar to those of mammal and are therefore widely used in studies of screening drugs have anti-inflammatory efficacy (Rodríguez-Ruiz et al., 2020). In the present study, DE reduced ROS production as well as neutrophil aggregation in CuSO_4_-stimulated zebrafish larvae, and DE also decreased the expression levels of iNOS, TNF-α, IL-6, and IL-10, thus inhibiting the inflammatory response in CuSO_4_-stimulated zebrafish larvae.

In summary, this study showed that DE reduced the morphological changes and NO production and played a bidirectional role in regulating ROS production in LPS-stimulated RAW264.7 cells. DE promoted the expression levels of IL-10 and CD206 in M2-type macrophages to suppress inflammatory reflections. And DE could reduce the apoptosis of RAW264.7 cells, which might be related to the fact that DE released the LPS-induced G0/G1 phase block. In addition, DE reduced CuSO4-stimulated ROS production as well as neutrophil aggregation and decreased the expression levels of iNOS, TNF-α, IL-6, and IL-10 in zebrafish larvae. Therefore, we conclude that DE reduces LPS-induced inflammatory response by regulating the polarization and apoptosis in RAW264.7 cells, and also reduces Cuso4-induced inflammatory response in zebrafish larvae.

## Data Availability

The original contributions presented in the study are included in the article/Supplementary Material, further inquiries can be directed to the corresponding authors.

## References

[B1] Aksentijevich M. Lateef S. S. Anzenberg P. Dey A. K. Mehta N. N. (2020). Chronic Inflammation, Cardiometabolic Diseases and Effects of Treatment: Psoriasis as a Human Model. Trends Cardiovasc Med. 30 (8), 472–478. 10.1016/j.tcm.2019.11.001 31837960 PMC7428846

[B2] Besson A. Dowdy S. F. Roberts J. M. (2008). CDK Inhibitors: Cell Cycle Regulators and beyond. Dev. Cell. 14 (2), 159–169. 10.1016/j.devcel.2008.01.013 18267085

[B3] Bjørn M. E. Hasselbalch H. C. (20152015). The Role of Reactive Oxygen Species in Myelofibrosis and Related Neoplasms. Mediat. Inflamm. 2015–11. 10.1155/2015/648090 PMC4619981 26538833

[B4] Blaser H. Dostert C. Mak T. W. Brenner D. (2016). TNF and ROS Crosstalk in Inflammation. Trends Cell. Biol. 26 (4), 249–261. 10.1016/j.tcb.2015.12.002 26791157

[B5] Chen J. Wu W. Zhang M. Chen C. (2019). Taraxasterol Suppresses Inflammation in IL-1β-induced Rheumatoid Arthritis Fibroblast-like Synoviocytes and Rheumatoid Arthritis Progression in Mice. Int. Immunopharmacol. 70, 274–283. 10.1016/j.intimp.2019.02.029 30851708

[B6] Daniel B. Nagy G. Czimmerer Z. Horvath A. Hammers D. W. Cuaranta-Monroy I. (2018). The Nuclear Receptor PPARγ Controls Progressive Macrophage Polarization as a Ligand-Insensitive Epigenomic Ratchet of Transcriptional Memory. Immunity 49 (4), 615–e6. e6. 10.1016/j.immuni.2018.09.005 30332629 PMC6197058

[B7] Davies L. C. Jenkins S. J. Allen J. E. Taylor P. R. (2013). Tissue-resident Macrophages. Nat. Immunol. 14 (10), 986–995. 10.1038/ni.2705 24048120 PMC4045180

[B8] Esatbeyoglu T. Obermair B. Dorn T. Siems K. Rimbach G. Birringer M. (2017). Sesquiterpene Lactone Composition and Cellular Nrf2 Induction of *Taraxacum officinale* Leaves and Roots and Taraxinic Acid β-d-Glucopyranosyl Ester. J. Med. Food 20 (1), 71–78. 10.1089/jmf.2016.0105 28026992

[B9] Feng B. Jiao P. Nie Y. Kim T. Jun D. van Rooijen N. (2011). Clodronate Liposomes Improve Metabolic Profile and Reduce Visceral Adipose Macrophage Content in Diet-Induced Obese Mice. PloS one 6 (9), e24358. 10.1371/journal.pone.0024358 21931688 PMC3171445

[B10] Ferrer M. D. Busquets-Cortés C. Capó X. Tejada S. Tur J. A. Pons A. (2019). Cyclooxygenase-2 Inhibitors as a Therapeutic Target in Inflammatory Diseases. Curr. Med. Chem. 26 (18), 3225–3241. 10.2174/0929867325666180514112124 29756563

[B11] Gong L. Yu L. Gong X. Wang C. Hu N. Dai X. (2020). Exploration of Anti-inflammatory Mechanism of Forsythiaside A and Forsythiaside B in CuSO4-Induced Inflammation in Zebrafish by Metabolomic and Proteomic Analyses. J. Neuroinflammation 17 (1), 173. 10.1186/s12974-020-01855-9 32493433 PMC7271515

[B12] Harusato A. Naito Y. Takagi T. Uchiyama K. Mizushima K. Hirai Y. (2013). BTB and CNC Homolog 1 (Bach1) Deficiency Ameliorates TNBS Colitis in Mice: Role of M2 Macrophages and Heme Oxygenase-1. Inflamm. Bowel Dis. 19 (4), 740–753. 10.1097/MIB.0b013e3182802968 23446334

[B13] Hernandez A. Patil N. K. Stothers C. L. Luan L. McBride M. A. Owen A. M. (2019). Immunobiology and Application of Toll-like Receptor 4 Agonists to Augment Host Resistance to Infection. Pharmacol. Res. 150, 104502. 10.1016/j.phrs.2019.104502 31689522 PMC6884699

[B14] Hong H. Y. Jeon W. K. Kim B. C. (2008). Up-regulation of Heme Oxygenase-1 Expression through the Rac1/NADPH oxidase/ROS/p38 Signaling Cascade Mediates the Anti-inflammatory Effect of 15-Deoxy-Delta 12,14-prostaglandin J2 in Murine Macrophages. FEBS Lett. 582 (6), 861–868. 10.1016/j.febslet.2008.02.012 18291107

[B15] Hu G. Wang J. Hong D. Zhang T. Duan H. Mu X. (2017). Effects of Aqueous Extracts of Taraxacum Officinale on Expression of Tumor Necrosis Factor-Alpha and Intracellular Adhesion Molecule 1 in LPS-Stimulated RMMVECs. BMC Complement. Altern. Med. 17 (1), 38. 10.1186/s12906-016-1520-3 28077102 PMC5225575

[B16] Huang M. H. Lin Y. H. Lyu P. C. Liu Y. C. Chang Y. S. Chung J. G. (2021). Imperatorin Interferes with LPS Binding to the TLR4 Co-receptor and Activates the Nrf2 Antioxidative Pathway in RAW264.7 Murine Macrophage Cells. Antioxidants (Basel) 10 (3), 362. 10.3390/antiox10030362 33673673 PMC7997471

[B18] Jiang S. H. Ping L. F. Sun F. Y. Wang X. L. Sun Z. J. (2016). Protective Effect of Taraxasterol against Rheumatoid Arthritis by the Modulation of Inflammatory Responses in Mice. Exp. Ther. Med. 12 (6), 4035–4040. 10.3892/etm.2016.3860 28101182 PMC5228288

[B19] Kikuchi T. Tanaka A. Uriuda M. Yamada T. Tanaka R. (2016). Three Novel Triterpenoids from *Taraxacum officinale* Roots. Molecules 21 (9), 1121. 10.3390/molecules21091121 PMC6274398 27618885

[B20] Kim Y. H. Choo S. J. Ryoo I. J. Ahn J. S. Yoo I. D. (2011). Eudesmanolides from Taraxacum Mongolicum and Their Inhibitory Effects on the Production of Nitric Oxide. Arch. Pharm. Res. 34 (1), 37–41. 10.1007/s12272-011-0104-5 21468913

[B21] Kimbrough D. Wang S. H. Wright L. H. Mani S. K. Kasiganesan H. LaRue A. C. (2018). HDAC Inhibition Helps Post-MI Healing by Modulating Macrophage Polarization. J. Mol. Cell. Cardiol. 119, 51–63. 10.1016/j.yjmcc.2018.04.011 29680681 PMC5991625

[B22] Lanzarin G. Venâncio C. Félix L. M. Monteiro S. (2021). Inflammatory, Oxidative Stress, and Apoptosis Effects in Zebrafish Larvae after Rapid Exposure to a Commercial Glyphosate Formulation. Biomedicines 9 (12), 1784. 10.3390/biomedicines9121784 34944599 PMC8698920

[B23] Lee H. W. Lee C. G. Rhee D. K. Um S. H. Pyo S. (2017). Sinigrin Inhibits Production of Inflammatory Mediators by Suppressing NF-Κb/MAPK Pathways or NLRP3 Inflammasome Activation in Macrophages. Int. Immunopharmacol. 45, 163–173. 10.1016/j.intimp.2017.01.032 28219839

[B24] Liang Y. Zha S. Tentaku M. Okimura T. Jiang Z. Ueno M. (2021). Suppressive Effects of Sulfated Polysaccharide Ascophyllan Isolated from Ascophyllum Nodosum on the Production of NO and ROS in LPS-Stimulated RAW264.7 Cells. Biosci. Biotech. Bioch 85 (4), 882–889. 10.1093/bbb/zbaa115 33580696

[B25] Lin X. Zhang J. Fan D. Hou J. Wang H. Zhu L. (2021). Frutescone O from Baeckea Frutescens Blocked TLR4-Mediated Myd88/NF-Κb and MAPK Signaling Pathways in LPS Induced RAW264.7 Macrophages. Front. Pharmacol. 12, 643188. 10.3389/fphar.2021.643188 33986676 PMC8112673

[B27] Liu M. Zhang C. Xu X. Zhao X. Han Z. Liu D. (2021). Ferulic Acid Inhibits LPS-Induced Apoptosis in Bovine Mammary Epithelial Cells by Regulating the NF-Κb and Nrf2 Signalling Pathways to Restore Mitochondrial Dynamics and ROS Generation. Vet. Res. 52 (1), 104. 10.1186/s13567-021-0097310.1186/s13567-021-00973-3 34256834 PMC8278735

[B28] Liu Z. Ren Z. Zhang J. Chuang C. C. Kandaswamy E. Zhou T. (2018). Role of ROS and Nutritional Antioxidants in Human Diseases. Front. Physiol. 9, 477. 10.3389/fphys.2018.00477 29867535 PMC5966868

[B30] Martinez M. Poirrier P. Chamy R. Prüfer D. Schulze-Gronover C. Jorquera L. (2015). *Taraxacum officinale* and Related Species-An Ethnopharmacological Review and its Potential as a Commercial Medicinal Plant. J. Ethnopharmacol. 169, 244–262. 10.1016/j.jep.2015.03.067 25858507

[B31] Mlcochova P. Winstone H. Zuliani-Alvarez L. Gupta R. K. (2020). TLR4-Mediated Pathway Triggers Interferon-independent G0 Arrest and Antiviral SAMHD1 Activity in Macrophages. Cell. Rep. 30 (12), 3972–e5. e5. 10.1016/j.celrep.2020.03.008 32209460 PMC7109521

[B32] Nathan C. Cunningham-Bussel A. (2013). Beyond Oxidative Stress: an Immunologist's Guide to Reactive Oxygen Species. Nat. Rev. Immunol. 13 (5), 349–361. 10.1038/nri3423 23618831 PMC4250048

[B33] Park C. M. Park J. Y. Noh K. H. Shin J. H. Song Y. S. (2011). *Taraxacum officinale* Weber Extracts Inhibit LPS-Induced Oxidative Stress and Nitric Oxide Production via the NF-Κb Modulation in RAW264.7 Cells. J. Ethnopharmacol. 133 (2), 834–842. 10.1016/j.jep.2010.11.015 21075189

[B34] Rodríguez-Ruiz L. Lozano-Gil J. M. Lachaud C. Mesa-Del-Castillo P. Cayuela M. L. García-Moreno D. (2020). Zebrafish Models to Study Inflammasome-Mediated Regulation of Hematopoiesis. Trends Immunol. 41 (12), 1116–1127. 10.1016/j.it.2020.10.006 33162327

[B35] Saini R. Singh S. (2019). Inducible Nitric Oxide Synthase: An Asset to Neutrophils. J. Leukoc. Biol. 105 (1), 49–61. 10.1002/JLB.4RU0418-161R 30285282

[B36] Sharifi-Rad M. Roberts T. H. Matthews K. R. Bezerra C. F. Morais-Braga M. F. B. Coutinho H. D. M. (2018). Ethnobotany of the Genus Taraxacum-Phytochemicals and Antimicrobial Activity. Phytother. Res. 32 (11), 2131–2145. 10.1002/ptr.6157 30039597

[B37] Shen S. Wang K. Zhi Y. Shen W. Huang L. (2020). Gypenosides Improves Nonalcoholic Fatty Liver Disease Induced by High-Fat Diet Induced through Regulating LPS/TLR4 Signaling Pathway. Cell. Cycle 19 (22), 3042–3053. 10.1080/15384101.2020.1829800 33121337 PMC7714522

[B38] Sica A. Mantovani A. (2012). Macrophage Plasticity and Polarization: *In Vivo* Veritas. J. Clin. Invest. 122 (3), 787–795. 10.1172/JCI59643 22378047 PMC3287223

[B40] Tu Y. J. Tan B. Jiang L. Wu Z. H. Yu H. J. Li X. Q. (2021). Emodin Inhibits Lipopolysaccharide-Induced Inflammation by Activating Autophagy in RAW 264.7 Cells. Chin. J. Integr. Med. 27 (5), 345–352. 10.1007/s11655-020-3477-9 32840732

[B41] Turner M. D. Nedjai B. Hurst T. Pennington D. J. (2014). Cytokines and Chemokines: At the Crossroads of Cell Signalling and Inflammatory Disease. Biochim. Biophys. Acta 1843 (11), 2563–2582. 10.1016/j.bbamcr.2014.05.014 24892271

[B42] Utzeri E. Usai P. (2017). Role of Non-steroidal Anti-inflammatory Drugs on Intestinal Permeability and Nonalcoholic Fatty Liver Disease. World J. Gastroenterol. 23 (22), 3954–3963. 10.3748/wjg.v23.i22.3954 28652650 PMC5473116

[B43] Walls M. Mehta P. P. Baxi S. M. Liu K. Li C. Smeal T. (2011). Abstract 4479: Targeting Non-small Cell Lung Cancer Cells Harboring a Pik3ca Mutation by a Novel and Oral Pi3k Selective Inhibitor, Pf-4989216. Cancer Res. 71 (8 Suppl. ment), 4479. 10.1158/1538-7445.AM2011-4479

[B44] Wang C. Z. Wang M. M. Wei Q. Shao B. X. Chen S. Zhang X. X. (2020). Simultaneous Determination of Four Index Components in Roots and Leaves of Herba Taraxaci by UPLC-MS/MS. Her. Med. 39 (10), 5. (Chinese). 10.3870/j.issn.1004-0781.2020.10.016

[B45] Wang Y. Han C. C. Cui D. Li Y. Ma Y. Wei W. (2017). Is Macrophage Polarization Important in Rheumatoid Arthritis? Int. Immunopharmacol. 50, 345–352. 10.1016/j.intimp.2017.07.019 28750350

[B46] Yang C. S. Kim J. J. Lee S. J. Hwang J. H. Lee C. H. Lee M. S. (2013). TLR3-triggered Reactive Oxygen Species Contribute to Inflammatory Responses by Activating Signal Transducer and Activator of Transcription-1. J. Immuno 190 (12), 6368–6377. 10.4049/jimmunol.1202574 23670194

[B47] Yi Y. S. (2016). Folate Receptor-Targeted Diagnostics and Therapeutics for Inflammatory Diseases. Immune Netw. 16 (6), 337–343. 10.4110/in.2016.16.6.337 28035209 PMC5195843

