# Supplementary figures and images for: Anti-Inflammatory Effects and Mechanisms of Dandelion in RAW264.7 Macrophages and Zebrafish Larvae
Source: Front Pharmacol. 2022 Aug 25;13:906927. doi: 10.3389/fphar.2022.906927 (PMC9454954; doi:10.3389/fphar.2022.906927)

Supplementary Figure 1

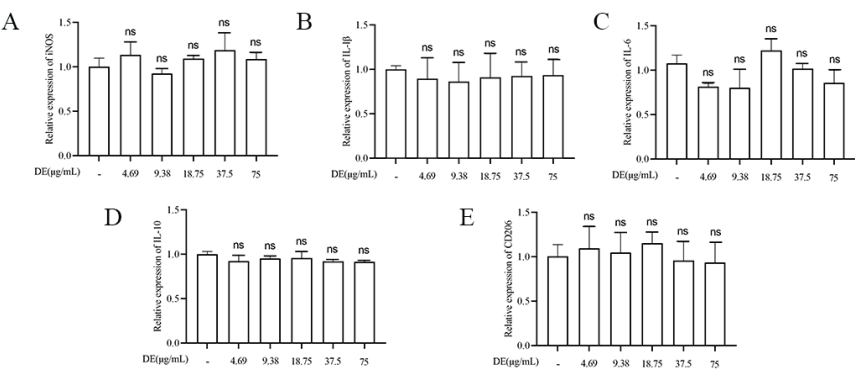

Supplementary Figure 2

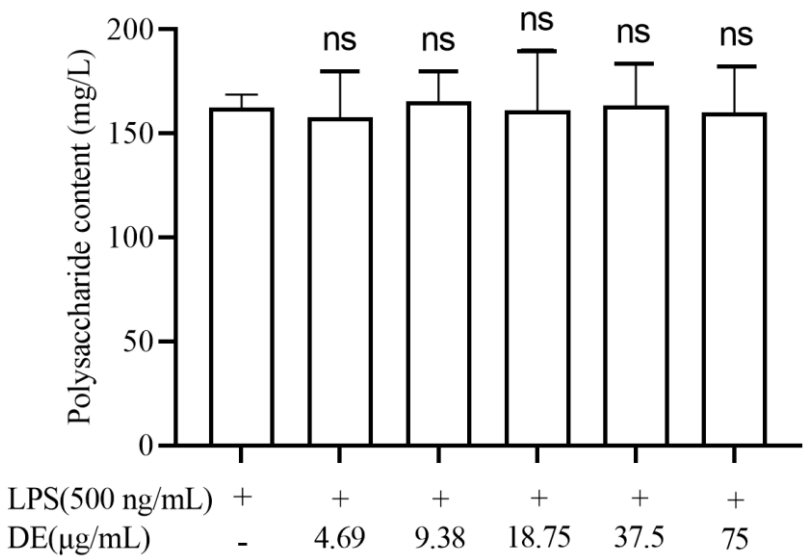

Supplementary Figure 3

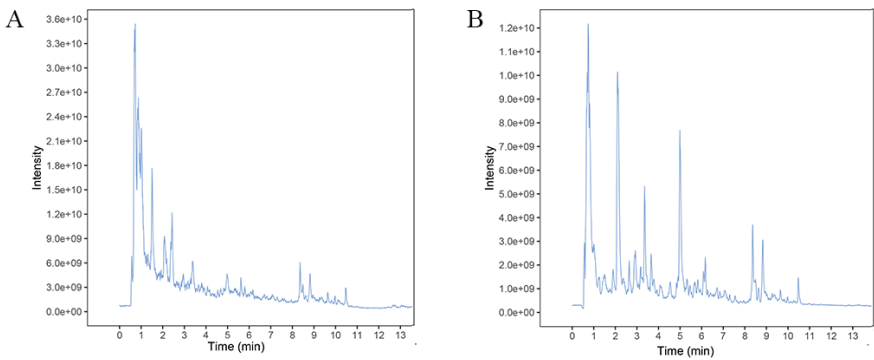

Supplement: Supplementary file 1 [file DataSheet1.PDF]
